# Robust Lane Sensing and Departure Warning under Shadows and Occlusions

**DOI:** 10.3390/s130303270

**Published:** 2013-03-11

**Authors:** Rodolfo Tapia-Espinoza, Miguel Torres-Torriti

**Affiliations:** Department of Electrical Engineering, Pontificia Universidad Católica de Chile, Vicuña Mackenna 4860, Casilla 306-22, Santiago, Chile; E-Mail: rntapia@puc.cl

**Keywords:** road sensing, lane detection and tracking, lane departure warning, mean-shift clustering, gabor filters, Gaussian Markov Random Fields, RANSAC

## Abstract

A prerequisite for any system that enhances drivers' awareness of road conditions and threatening situations is the correct sensing of the road geometry and the vehicle's relative pose with respect to the lane despite shadows and occlusions. In this paper we propose an approach for lane segmentation and tracking that is robust to varying shadows and occlusions. The approach involves color-based clustering, the use of MSAC for outlier removal and curvature estimation, and also the tracking of lane boundaries. Lane boundaries are modeled as planar curves residing in 3D-space using an inverse perspective mapping, instead of the traditional tracking of lanes in the image space, *i.e.*, the segmented lane boundary points are 3D points in a coordinate frame fixed to the vehicle that have a depth component and belong to a plane tangent to the vehicle's wheels, rather than 2D points in the image space without depth information. The measurement noise and disturbances due to vehicle vibrations are reduced using an extended Kalman filter that involves a 6-DOF motion model for the vehicle, as well as measurements about the road's banking and slope angles. Additional contributions of the paper include: (i) the comparison of textural features obtained from a bank of Gabor filters and from a GMRF model; and (ii) the experimental validation of the quadratic and cubic approximations to the clothoid model for the lane boundaries. The results show that the proposed approach performs better than the traditional gradient-based approach under different levels of difficulty caused by shadows and occlusions.

## 1. Introduction

According to the World Health Organization (WHO), about 1.2 million people get killed in traffic accidents each year worldwide, while the number of injured is estimated to be 50 million. On the other hand, the cost of road accidents to national economies has been estimated to roughly correspond to 1% of their gross national product for low-income countries, 1.5% for middle-income countries and 2% for high-income countries, accruing to an estimated global of over US$ 518 billion each year [1]. In this context, the development of driver assistance systems (DAS) that address the main risk factors and collision causes is essential to further reduce the number of road accidents.

Different traffic control, road safety and driver assistance systems have been proposed and developed during the last decade; see recent surveys in [2–4]. Some DAS focus on the driver, employing biometric measures of the driver's performance like alert state and fatigue and awareness levels [5,6]. Some others focus on traffic monitoring and control schemes [2,7] to improve road safety. A third group aims at increasing situational awareness with different sensing technologies, mainly combining visible spectrum cameras with image processing and computer vision techniques [4,8,9]. Among the different road and traffic perception systems, lane recognition is a prerequisite for lane departure warning and a fundamental enabler for advanced DAS [10,11]. However, most of the existing vision-based approaches rely mainly on the analysis of the spatial gradient of the road image to extract lane boundaries. The main drawback of these methods is that if the road structure is not regular or well-delimited, or if there are shadows and occlusions, then it may not be easy to extract the edges correctly and reliably. In this paper, we propose a vision-based lane detection and tracking method capable of estimating the lane geometry and relative vehicle position even if lane boundaries are not clear due to shadows, changes in illumination or partial occlusions caused by other vehicles. The paper also aims at answering some practical issues that are not conclusively addressed in the existing literature, which can be summarized in the following questions: Is textural information of the pavement useful for segmenting the road in a more robust way than classic schemes that rely on edge detection using gradient computations? Is color-based clustering reliable enough for segmenting the pavement region despite changes in hue and intensity? Can the complex clothoid road models be simplified? Is a quadratic or cubic polynomial approximation to the clothoid model good enough? How can the edge geometry be estimated when there are broken (discontinuous) edges due to occlusions, shadows, changes in illumination or lack of contrast? In prior approaches, textural features were not considered due to their higher computational cost, which limited their application on older and less powerful processors. However, very recent work [11] shows an increasing interest on texture. More specifically, the approach proposed in [11] relies on a cost function to label drivable and non-drivable road regions. The cost function takes into account ground plane discontinuities and texture descriptors based on Markov random fields to improve the robustness of the drivable region segmentation.

The proposed approach first segments the area corresponding to the road employing hue-intensity clustering and textural features. Using the segmented pavement region, and inverse perspective mapping and the MSAC variant of the RANSAC algorithm, the lane geometry and position relative to the vehicle in 3D coordinates is obtained. The estimation of the lane geometry and vehicle is further improved with an extended Kalman filter (EKF) applied to the features in 3D space taking also into account the motion model of the vehicle. It is to be noted that the lane boundaries are modeled as curves contained in a 2D plane residing in 3D space. This allows to take advantage of computationally simple homography transformations between the planar road model and the imaging sensor plane. However, the dynamic model of the vehicle takes into account the slope and bank angle of the road measured with a gyroscope. Even if the proposed road model only takes into account the curvature of the road in the plane tangent to the vehicle's wheels and does not consider the road's geodesic and torsional curvature in the standard Darboux frame formulation, the proposed system is compared with previous methods and shown to be robust under a wide range of conditions including quality of lane marks (if they exist), lighting conditions and road occlusion by other vehicles. The proposed lane sensing system should help to enhance the safety of drivers and pedestrians by preventing unintended lane changes due to distracted driving or reducing risky maneuvers due to excessive speed for a given lane curvature. Another contribution of this paper is the comparison of the textural features considered, which were generated with two textural models: (i) Gabor features; (ii) a Gaussian Markov random field model. Textural features have not been exploited enough due to their computational cost and the lack of computational power in the past, but are an important aspect for making the road segmentation more robust under low illumination levels. A more detailed review of the existing vision-based approaches to lane detection is presented in the next section. The remainder of the paper is organized as follows. Section 3 explains the proposed approach. The testing methodology and results are presented in Section 4, followed by the conclusions in the last section. The paper summarizes several results in the lane detection literature, which complement the recent surveys in [10,12], and presents a clear derivation of the Inverse Perspective Mapping and lane model equations in Section 3.3. The proposed approach relies on well-known mathematical tools or models, some of which have been employed in previous work in the field of lane detection, and thus this article should also have a reference and tutorial value.

## 2. Existing Approaches

The main sensing devices that have been proposed for lane detection and tracking include radars [13–15], laser range finders [16,17], active infrared sensors that detect lane crossings by measuring the IR LED light reflected by the road markings [18,19], and standard visible spectrum perspective cameras [10,12]. Although radar and laser based sensors are very good at detecting the road boundaries (curbs, berms) as well as other vehicles, regardless of the illumination conditions, their major drawbacks are their elevated cost compared with the alternative sensing modalities and the fact that lane markings only have color information and no 3D structure that could be obtained by the volume measuring sensors. On the other hand, reflective IR sensors only work close to the lane markings and have no real look-ahead capability. Because of these disadvantages, cameras are the most frequently employed modality for lane and road sensing. Some lane detection and tracking systems employ multiple cameras to perform stereo imaging and obtain 3D information or combine different sensing devices [11]. However, since human drivers are able to process adequately visual data under a wide range of driving conditions without requiring 3D information, here we will focus on the monocular vision-based approach.

Most vision-based approaches for lane detection extract lane edges and geometry information using gradient thresholding [20–22] and improvements relying on steerable filters for detecting solid-line and segmented-line marks [12]. Some other approaches have proposed maximizing the likelihood of deformable shape models of the road [23] and similar template matching methods [24]. The use of color, gray-scale intensities and textural cues of the pavement has also been suggested in the past, however, using color information or pixel intensities alone is not sufficient because this information varies significantly with illumination and environmental conditions. For this reason, the first approaches introduced simple texture descriptors based on local covariance matrices of the pixel intensities [25,26] or neural network classifiers [27]. More recently, Cheng *et al.* [28] use lane marking color cues for structured roads, but apply mean-shift for segmenting unstructured roads. Other recent approaches have also considered Gabor features with *k*-means clustering [29].

In order to cope with road variability and render lane estimation more robust to external disturbances (e.g., illumination, visibility, lack of road structure), lane position tracking can incorporate Kalman filters and sensor fusion techniques based on the vehicle's state monitoring [15] and GPS measures [30]. Some researchers have also considered the importance of using computationally simpler but robust road models, for example, Ruyi *et al.* [31] propose an approach that efficiently combines a modified distance transform to obtain the road edges' location together with a particle filter to track the edges' information. The reader is referred to [10,12] for surveys on the recent progress in lane detection and tracking. We complement these surveys with a brief summary presented in Table 1.

## 3. Proposed Lane Detection and Tracking Approach

The proposed approach involves the following main steps, which are explained in more detail in the following sections. First, the pavement area is segmented using the mean-shift clustering approach or employing color and texture information together with a multivariate Gaussian classifier. Standard morphological operations are applied to the segmentation in order to remove small isolated regions that do not belong to the pavement area and fill small holes in the pavement area produced by misclassified pixels. In the second step, the edges obtained from the segmented pavement are projected back onto the road plane in 3D world coordinates using an inverse perspective projection. The parameters of the curves that model the road boundaries are obtained using the MSAC variant of the RANSAC algorithm [32]. An Extended Kalman filter (EKF) is employed to track the lane position in 3D coordinates. Finally, a rule-based approach is used to estimate lane departure situations. The results of the lane detection and tracking can be projected from world coordinates to the image plane, thus enhancing the driver's understanding of the road scene and providing a visual confirmation of the system's performance.

### 3.1. Road Segmentation

Three different methods were implemented to segment the road: A color clustering approach employing the mean-shift algorithm and two approaches using color and texture features modeled as the response energies of a bank of Gabor filters or as the parameters of a Gaussian Markov random field (GMRF). These three methods are briefly explained next.

#### 3.1.1. Mean Shift Clustering

Mean shift is an iterative non-parametric feature-space analysis technique [39], widely employed in computer vision and image processing for finding modes in the distribution of certain data of a feature-space, *i.e.*, find the local maxima of the underlying probability density function from the available data without actually estimating the density In other words, mean-shift considers the feature space as an empirical probability density function and the data as samples drawn from the underlying probability distribution. Considering a finite set of samples (imageS) ⊂ ℝ*^n^* and a weighting function (kernel) *K* : *x* ∈ ℝ*^n^* → *K*(*x*) ∈ ℝ_+_, with *∫*_ℝn_*K*(*x*)*dx* = 1, *K*(−*x*) = *K*(*x*). If the kernel is a function with compact support on a neighborhood of the origin, often the closed ball *N_r_*(*x*) = {*w* ∈ ℝ^n^ : ║*w* − *x*║ ≤ *r*} of radius *r* and centered at *x*, the weighted mean at *x* of the samples is then

$$m(x)=\frac{\sum_{s_{i}\in N_{r}(x)}K(s_{i}-x)s_{i}}{\sum_{s_{i}\in N_{r}(x)}K(s_{i}-x)}$$

The repeated movement of data points *s_i_* ∈ (images) to sample means *m*(*s_i_*) is the so-called *mean-shift algorithm*, *i.e.*, in each iteration *s* ← *m*(*s*) is performed for all *s* ∈ (imageS) simultaneously until the means converge [39].

**Figure 1.**
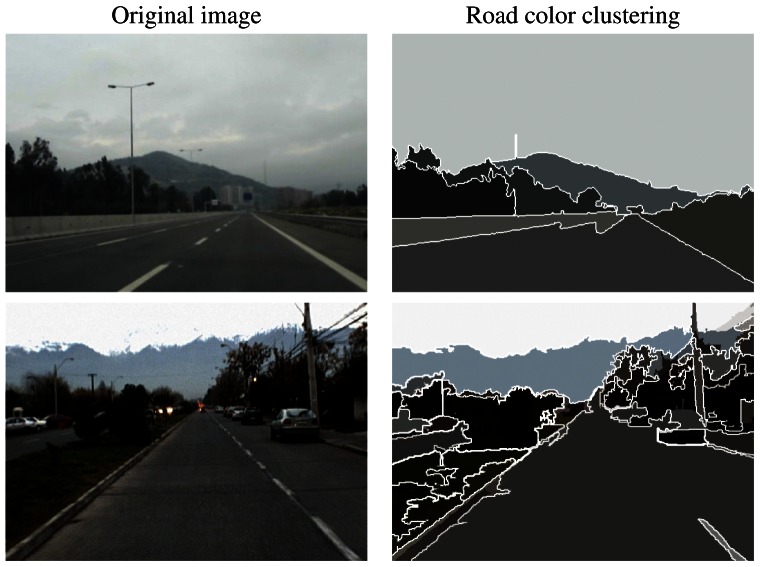
Example results from the mean-shift segmentation using bandwidth parameters *h_s_* = 2, *h_r_* = 4.

Our approach to the segmentation of the road pavement area is based on the mean-shift algorithm proposed in [40], which considers a feature space composed of spatial domain feature *x^s^* in the image coordinate system and intensity values *x^r^* in the range domain of the L*u*v* color space. The kernel considered is a multivariate function defined as the product of two radially symmetric kernels:

(1) $$K_{h_{s},h_{r}}(x)=\frac{C}{{h_{s}}^{2}{h_{r}}^{p}}k\left({\left\|\frac{x^{s}}{h_{s}}\right\|}^{2}\right)k\left({\left\|\frac{x^{r}}{h_{r}}\right\|}^{2}\right)$$

where *h_s_* and *h_r_* are respectively the spatial bandwidth and color range parameters, *p* is the number of channels of the color domain, *C* is a normalization constant and $k:u\in \mathbb{R}\to \frac{3}{4}(1-u^{2})\cdot 1_{\left\{u:\left|u\right|\leq 1\right\}}\subset \mathbb{R}$ is the Epanechnikov kernel. The final segmentation is obtained by grouping all pixels that are closer than *h_s_* in the spatial domain and closer than *h_r_* in the range domain, as shown in the example results of Figure 1.

#### 3.1.2. Gabor and GMRF Texture Features

The second approach implemented for road segmentation is based on the classification of texture and color features using a standard multivariate Gaussian classifier (MVGC) [41]. The texture features associated to each pixel are generated either as the response to a bank of Gabor filters, or as the parameters of a GMRF [42]. To improve the identification of pavement and other elements, RGB color components of the Gaussian filtered image are also included into the feature vectors.

**Figure 2.**
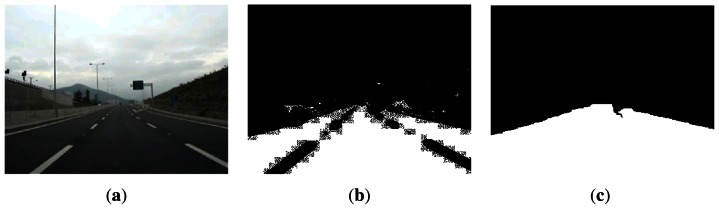
Example of road segmentation using Gabor textures. (**a**) Original image; (**b**) Gabor-based texture segmentation; (**c**) Morphological opening and closing.

The impulse response of the Gabor filter in the spatial domain coordinates can be expressed as

$$h(p|\sigma ,\lambda ,\theta ,\phi ,p_{0})=\exp\left\{-\frac{\left\|p-p_{0}\right\|}{2\sigma^{2}}\right\}\cdot \sin\left(\frac{2\pi }{\lambda }\left({\left[p-p_{0}\right]}^{T}\left[\begin{array}{c} \cos\theta \\ \sin\theta \end{array}\right]\right)+\phi \right)$$

where *p* = [*x y*]*^T^* is the spatial coordinate, λ corresponds to the wavelength of the sinusoid, *σ* is the spread of the Gaussian envelope, *θ* and *ϕ* are respectively the orientation and phase of the Gabor filter, and *p*_0_ is the center of the neighborhood *N*(*p_0_*) on which the truncated response is defined (the region of the filter mask). In order to discriminate different textures correctly regardless of the textures phase, the total response energy of a pair of filters in quadrature phase relationship with *ϕ* = {0, π/2} is often employed. Quadrature filters are motivated by the fact that odd Gabor masks resemble edge detectors, while even masks act as blob detectors. The total response energy is obtained from the convolution of *h*(·) with the image as *E^2^*(*σ*, *λ*, *θ*∣*p*) = Σ_*ϕ*={0, π/2}_ {Σ_*p*∈*N*(*po*)_ *h*(*p*∣*σ*, *λ*, *θ*, *ϕ*, *p0*)*I*(*p*)}^2^ , where *I* : Ω ⊂ ℕ^2^ → ℤ+, *p* ↦ *I_k_*(*p*) for *p* ∈ Ω, is the intensity function representing the image. Using the response energies at different wavelengths λ and orientations *θ*, it is possible to construct the texture descriptor at pixel *p* as the feature vector *X_G_*(*p*) = {*E^2^*(λ_1_, *θ*_1_∣*s*) ⋯ *E*^2^(λ_i_, θj∣s*)* ⋯ *E^2^*(*λ_n_, θ_m_*∣*s*) *s_r_*(*p*) *s_g_*(*p*) *s_b_*(*p*)}*^T^* ∈ ℝ^m·n+3^, where *s_r_, s_g_, s_b_* are the responses of the red, green and blue channels to a Gaussian filter, and the dependency on *σ* has been suppressed by making *σ* a constant or dependent on λ [42]. Figure 2 shows an example of road pavement segmentation obtained using the Gabor textural features, and the posterior morphological opening and closing operations, whose purpose is to remove the small isolated areas and fill small holes in the region classified as pavement.

An alternative description of textural properties is possible in terms of the parameters of Gaussian Markov random fields (GRMF) [42], which describe the statistical dependency between the intensity *I*(*p*) at pixel *p* and that of its neighbors with the equation *I*(*p*) *=* Σ*_r_*_∈_*_∆N_θ_r_* (*I*(*p* + *r*) + *I*(*p* − *r*)) + *e*(*p*), where ∆*N* = {*r* : *p* ± *r* ∈ *N*(*p*)} for some neighborhood *N*(*p*) of *p* and *e*(*p*) is zero-mean Gaussian noise. Here it is assumed that the observations *I*(*p*) are zero-mean Gaussian, and that the dependency of the intensity at the central pixel *p* is symmetric with respect to the intensities of pixels in the neighborhood *N*(*p*), *i.e.*, *θ_r_* = *θ*_−*r*_, ∀ *r* ∈ *N*(*p*).

A consistent and computationally efficient estimate of the parameters Θ = [*θ*_*r*1_ *θ*_*r*2_ ⋯ *θ*_*r_n_*_]^T^, *r_i_* ∈ ∆*N* can be obtained applying the least squares method as

$$\Theta *={\left[\sum_{p\in \Omega_{I}}q(p)q{\left(p\right)}^{T}\right]}^{-1}\left[\sum_{p\in \Omega_{I}}q(p)I(p)\right]$$

where Ω*_I_* is an interior region of the image and

$$q(p)\overset{def}{=}\left[\begin{array}{c} I(p+r_{1})+I(p-r_{1})\\ I(p+r_{2})+I(p-r_{2})\\ \vdots \\ I(p+r_{n})+I(p-r_{n}) \end{array}\right],r_{i}\in \Delta N$$

The estimate of the noise variance can be calculated as $\nu *=\frac{1}{\left|\Omega_{I}\right|}\sum_{p\in \Omega_{I}}{\left(I(p)-\Theta {*}^{T}q(p)\right)}^{2}.$

Previous work by the authors [42] has shown that textures can also be adequately characterized by feature vectors defined in terms of the estimated GMRF parameters as *X_M_* = {Θ* υ*/*ρ^2^*}*^T^* , where *ρ* is the sample variance of the texture as a feature vector {Θ* v*}*^T^* .

### 3.2. Lane Boundaries Extraction

Once the road pavement areas have been segmented, the lane boundaries are obtained computing the spatial gradient directly on the segmented image. This is key to making the approach more robust to shadows and occlusions than other methods that extract lane boundaries by computing the spatial gradients directly on the pixel intensities of the original image. To compute spatial gradients, here we employ steerable filters because they are computationally efficient and, as shown in [12], can reliably detect continuous and segmented line markings under different road conditions. On the other hand, steerable filters directly applied to the original image as proposed in [12] provide a benchmark method against which we can compare ours.

Steerable filters correspond to oriented LoG filters calculated using three precomputed second-order derivative masks, one in the horizontal direction, *G_uu_*, one in the vertical direction, *G_vv_*, and one in the diagonal direction, *G_uv_*, allowing an efficient calculation of the oriented filter *G_θ_* in any direction *θ* according to *G_θ_* (*u,v*) = *G_uu_* cos^2^(*θ*) + *G_vv_sin^2^*(*θ*) + *G_uv_cos*(*θ*) *sin*(*θ*); (see [43] for further details). In order to maximize the response of the steerable filters to the lane marks and road boundaries, a bank composed by two filters is applied to the road area extracted in the previous step. One filter is oriented along the normal of the expected left boundary of the lane, while the other along the normal of the expected right boundary. The thresholded responses to both filters are then combined into one unique response applying the logical “or” operator. The orientation of both filters is updated in each iteration according to the orientation of the lane's boundaries that were obtained in the previous iteration.

To further improve the extraction of boundaries and lane markings, undesired steerable filter responses produced by potholes, cracks and other spots in the image are removed if the segments do not satisfy: (i) a minimum length criterion; or (ii) a geometric criterion which measures the similarity of the segment to a curve. The geometric criterion is based on the fact that a straight line segment represents the degenerate case of an ellipse whose eccentricity *e* = *f*/a (the ratio between its focal length *f* and its major semi-axis a) is one. Hence, it is possible to discard all segments of connected pixels for which their approximating ellipse does not satisfy *e* > δ_1_, for some *δ* ∈ (0, 1), and their length is not greater than a minimum length *δ_2_*. The parameters *δ_1_* and *δ_2_* can be estimated from the standard road geometry, average lane marking lengths and perspective considerations. Fitting ellipses to segments of connected pixels can be quickly done by computing the second moment of the group of pixels. This is a rather common approach to find the major/minor axes of the ellipsoid enclosing a cloud of points, considering that level-sets of a multivariate Gaussian distribution are ellipsoids, and that the ellipsoid axes are oriented along the eigenvectors of the covariance matrix, while the magnitude of the axes is given by the corresponding eigenvalues [44].

**Figure 3.**
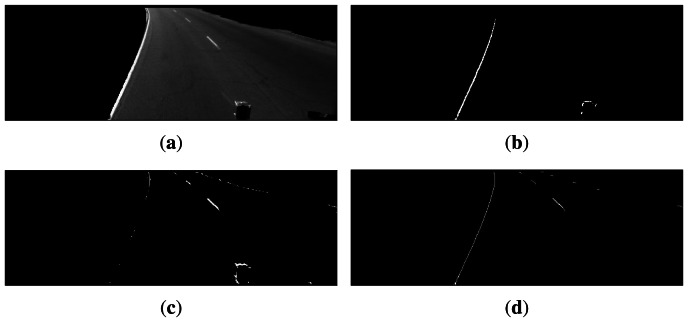
Application of steerable filters, and the edge length and eccentricity selection criteria to a road image of the Caltech Lanes dataset [45]. (**a**) Segmented road area using Gabor textures; (**b**) Response to steerable filter with $\theta =-\frac{\pi }{6}$ after thresholding; (**c**) Response to steerable filter with $\theta =+\frac{\pi }{6}$ after thresholding; (**d**) Logical “or” operation of (**b**) and (**c**), and posterior edge selection based on the segment length and eccentricity criteria.

An example of the results obtained when applying the steerable filters to the segmented road pavement of Figure 3(a) is shown in Figure 3(b and c), with filter orientations *θ* = − π/6 and *θ* = +π/6, respectively. Once the logical “or” of the thresholded responses to the two oriented filters is computed, the edge selection criteria are applied to obtain the final edges shown in Figure 3(d), from which the lane geometry will be estimated.

The line segments extracted using the steerable filters and the geometric selection criteria must be labeled as left-boundary, right-boundary or center lane-markings. To this end, the Hough transform [46], ℋ : *I_s_*(*p*) → *H*(*ρ*, *θ*), of the segments image *I_s_*(*p*) is computed and the centroids of the three largest local maxima in the Hough matrix *H*(*ρ*, *θ*) are calculated, thus yielding the (*ρ*, *θ*) parameters of the major lines in the road. Segments of connected pixels are selected or discarded depending on how close their (*ρ*, *θ*) parameters are to the major lines. This process allows to further reduce the amount of local lines that do not correspond to actual lane boundaries. On the other hand, the principal (*ρ*, *θ*) parameters provide initial estimates for the lane position and geometry computation as will be explained in the next section. Furthermore, tracking and predicting the lane position in the next frames can be done efficiently in the (*ρ*, *θ*) parameter space using the Kalman filter instead of tracking the set of all salient points in the image.

### 3.3. Lane Geometry and Position Computation

Computing the lane geometry and position requires to fit some mathematical model of the road to the boundary segments extracted in the previous steps. To this end, it is convenient to project the segments in the image back from the camera's optical plane to the plane of the road. This process is often referred to as the Inverse Perspective Mapping (IPM) and allows to generate a top-view of the road [47].

**Figure 4.**
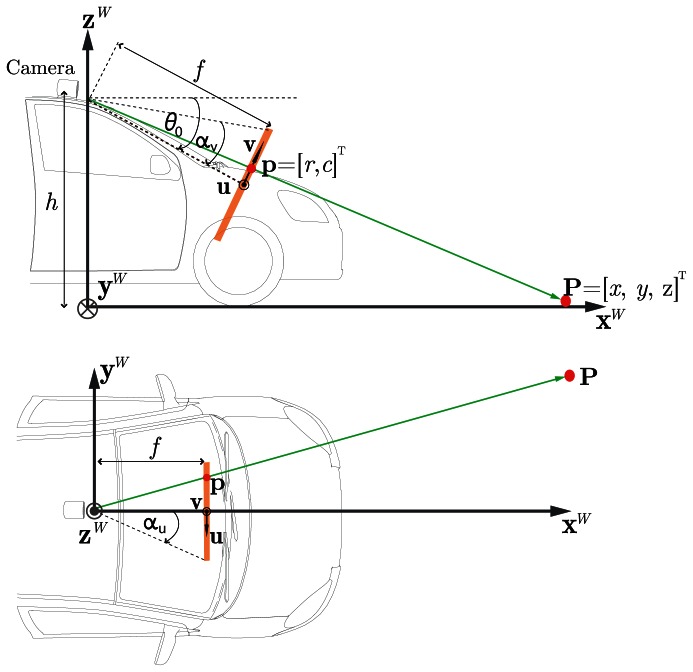
Inverse perspective projection model: point p on the optical plane is projected back onto point P on the road.

We construct the IPM using the standard pinhole camera model under the following assumptions:

(i) the world coordinate system $S_{W}\overset{\text{def}}{=}\{x^{W},y^{W},z^{W}\}$ is fixed to the vehicle (see Figure 4),
(ii) the camera is mounted on the vehicle at some given constant height *h* with respect to the ground, and tilted about its focal point in such a way that its optical axis forms an angle *θ*_0_ with respect to an axis parallel to x*^W^* passing through the focal point.
(iii) the road ahead the vehicle is locally flat in the first ˜100 m, *i.e.*, has negligible curvature with respect to the ground tangent plane at the vehicle's current position. This means the lane boundaries are modeled as curves contained in a 2D plane which is tangent to the wheels of the vehicle. However, it is to be noted that the plane has a slope angle *θ*_s_ and bank angle *θ_b_*. While these angles are not relevant to the IPM calculations, they are considered in the dynamic model of the vehicle employed in the implementation of the extended Kalman filter explained in Section 3.4.

For clarity of exposition, the plane containing point p shown in Figure 4 is an enlarged version of the camera's optical plane array, whose size is assumed to be *m* × *n* pixels large with *m* and *n* odd. The point p can be expressed as a coordinate pair p = (*u*, *v*) in the camera's reference system (imagesc) = {*u*, *v*}, where u is the camera's horizontal axis, while v is the camera's vertical axis. The origin of (imagesc) is centered on the optical plane at the intersection with the optical axis. The position of p can also be expressed in terms of the standard sensor's array or image row-column coordinate pair (*r*, *c*) ∈ ℕ^2^ and the pixel size *μ* (m/pixels). The expressions relating the coordinates (*u*, *v*) and (*r*, *c*) are thus given by:

(2) $$\nu (r)=\mu \left[\frac{m+1}{2}-r\right]\Rightarrow r(\nu )=\frac{m+1}{2}-\nu \mu^{-1}$$

(3) $$u(c)=\mu \left[c-\frac{n+1}{2}\right]\Rightarrow c(u)=\frac{n+1}{2}+u\mu^{-1}$$

As shown in Figure 4, the point p = (*u*, *v*) with associated coordinates (*r*, *c*) can be projected back onto a point P on the road with coordinates (*x*, *y*, 0) in (imagesw), *i.e.*, projected onto the plane $\bar{{\text{x}}^{W}{\text{y}}^{W}}$, using Equations (2) and (3) and projective geometry. The coordinates *x* and *y* of P are given by the following *inverse perspective mapping* (IPM) equations:

(4) $$x(r)=h\left(\frac{1+\left[1-2\left(\frac{r-1}{m-1}\right)\right]\tan(\alpha_{\nu })\tan(\theta_{0})}{\tan(\theta_{0})-\left[1-2\left(\frac{r-1}{m-1}\right)\right]\tan(\alpha_{\nu })}\right)$$

(5) $$y(r,c)=h\left(\frac{\left[1-2\left(\frac{c-1}{n-1}\right)\right]\tan(\alpha_{u})}{\sin(\theta_{0})-\left[1-2\left(\frac{r-1}{m-1}\right)\right]\tan(\alpha_{\nu })\cos(\theta_{0})}\right)$$

where *α_u_* and *α_v_* correspond to half of the horizontal and vertical angular field of view of the camera, and *θ*_0_ is the camera tilt. If the camera lens has a focal distance *f*, then tan(*α*_u_) = *u*(*n*)/*f* = (*n* − 1)*μ*/(2*f*) and tan(*α_v_*) = *v*(1)/*f* = (*m* − 1)*μ*/(2*f*).

Similarly, any point **P** = (*x*, *y*, 0) on the road (with coordinates expressed in (imagesw)) can be projected onto a point p on the optical plane array with associated coordinates (*r*, *c*) given by:

(6) $$r(x)=\frac{m-1}{2}\left[1+\frac{h-x\tan(\theta_{0})}{\left(h\tan(\theta_{0})+x)\tan(\alpha_{v}\right)}\right]+1$$

(7) $$c(x,y)=\frac{n-1}{2}\left[\frac{y}{\left(h\sin(\theta_{0})+x\cos(\theta_{0}))\tan(\alpha_{u}\right)}\right]+1$$

Equations (6) and (7) are useful for projecting on the image the computed boundaries and providing the driver with a visual confirmation of the operation of the system.

**Figure 5.**
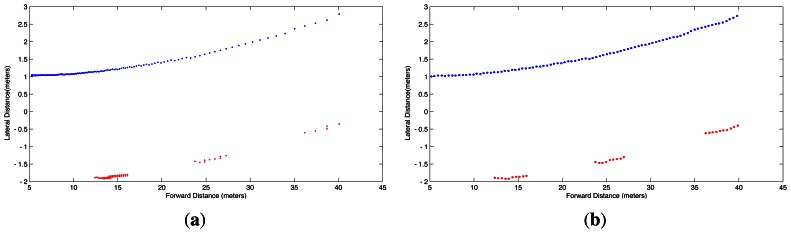
IPM Equations (4) and (5) applied to lane boundaries of Figure 3(d). (**a**) IPM of lane boundaries without interpolation; (**b**) IPM of lane boundaries after equispaced interpolation.

Due to the nonlinear form of Equations (4) and (5), evenly spaced increments in *r* and *c* do not produce evenly spaced points (*x*, *y*, 0) on the road plane as illustrated in Figure 5(a), which shows the top view of the left boundary and lane markings of the road lane in Figure 3(d). In other words, pixels covering the horizon represent larger distances of the road than pixels covering the pavement close to the car. In order to correct this situation that complicates fitting the model, evenly spaced interpolation is performed to ensure a uniform distribution of points per unit of length in the IPM image of the lane boundaries. The equispaced interpolation is shown in Figure 5(b).

The lane boundary points (*x*, *y*) on the ground plane $\bar{x^{W}y^{W}}$ must be fitted to a mathematical model of the road in order to determine the lane position relative to the world reference system (imagesw). The standard mathematical model assumes the curves are designed to produce a gradual transition from a straight section with infinite curvature radius to another straight section oriented in a different direction by linearly increasing and decreasing the curvature, thus preventing sudden changes in centrifugal forces. Mathematically, the linear increase/decrease in curvature is represented by segments of the Euler spiral, often called spiral transition curve or clothoid [48,49].

The normalized clothoid curve $\left(\sqrt{2R_{0}s_{0}}=1\right)$ can be approximated by a parametric representation in polynomial form after replacing the terms cos(*s*^2^) and sin(*s*^2^) by their power series expansion and integrating, thus yielding

(8) $$x(L)=\sqrt{2R_{0}s_{0}}\int_{0}^{L}\cos(s^{2})ds=L-\frac{L^{5}}{5.2!}+\frac{L^{9}}{9.4!}-\frac{L^{13}}{13.6!}+\cdots$$

(9) $$y(L)=\sqrt{2R_{0}s_{0}}\int_{0}^{L}\sin(s^{2})ds=\frac{L^{3}}{3}-\frac{L^{7}}{7.3!}+\frac{L^{11}}{11.5!}-\frac{L^{15}}{15.7!}+\cdots$$

where *R*_0_ corresponds to the radius of the circular curve at the end of the spiral and *L* the length of the spiral curve. The third order power series truncation is a practical approximation in road designs, which is amenable for simpler and faster computation of the road geometry. Hence, we approximate the clothoid model Equations (8) and (9) by a cubic polynomial:

(10) $$y(x)=\alpha_{3}x^{3}+\alpha_{2}x^{2}+\alpha_{1}x+\alpha_{0}$$

**Figure 6.**
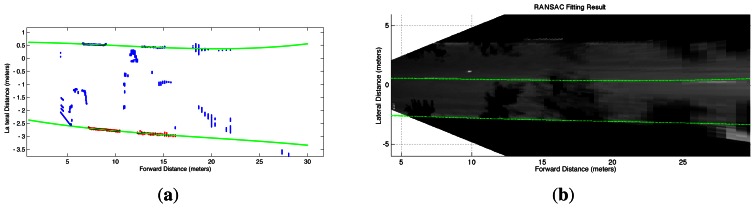
MSAC cubic polynomial fitting (green) in a street with many tree shadows. (**a**) Curves fitted to the extracted lane boundaries segments (top-view); (**b**) Lane boundaries superimposed on the segmented street pavement (top-view).

The parameters *α_i_*, *i* = 0, 1, 2, 3, for the lane boundary model Equation (10) can be robustly estimated despite occlusions and shadows using the MSAC (M-estimator Sample And Consensus) algorithm [50], which is a variation of the standard RANSAC algorithm [32] that weights the inliers according to how well they fit to a model, while the outliers are given a constant weight, thus improving the robustness of the estimation with no additional computational cost [51]. The application of the MSAC algorithm is presented in Figure 6, which shows the fitted polynomials (green continuous curves) despite the notoriously large amount of outliers caused by tree shadows. It is possible to notice in Figure 6(b) that the cubic polynomial boundaries are correctly located on the right lane of the segmented street, even if the connected pixels extracted for the lane's right boundary are very few (red segments in Figure 6(a)).

**Figure 7.**
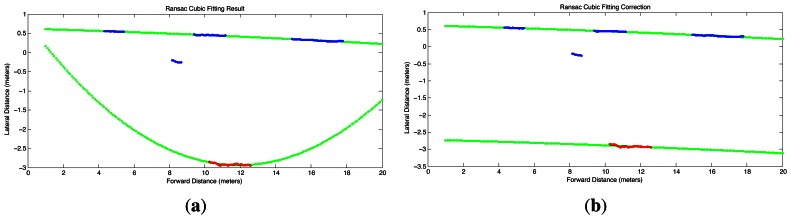
Example of the correction procedure for erroneous MSAC fitting results. (**a**)Erroneous MSAC fitting due to severe occlusion of the lane's right-boundary (red); (**b**)Correction of the lane's estimated right-boundary by vertically shifting the lane's estimated left-boundary (blue) and fitting it to the points of the right-boundary (red).

Sometimes the amount of occlusions and shadows can be even more severe than that of Figure 6 and the MSAC algorithm yields incorrect parameters for the polynomial. An example of an extreme situation is shown in Figure 7(a), in which the right boundary has to be estimated from a very short segment of connected pixels. This short segment leads MSAC to produce a right-boundary that is inconsistent with that obtained with the short edge segments of the left-boundary. To verify the reliability of the curves fitted with the MSAC algorithm, we implemented an additional step which checks the distance interval covered by the points in the consensus set along the longitudinal axis x*^w^*, as well as the number of points in the consensus sets. It was experimentally found that reliable boundaries could be identified whenever the points in the consensus set covered a distance at least between 5 m and 15 m, and the number of points was larger than 20% of total number of points that would be available if the edge would have been fully segmented. If these conditions are not fulfilled for only one of the lane boundaries and the mostly occluded boundary has a non-empty consensus set, then the correctly estimated curve for the mostly visible boundary with parameters $\alpha_{1}^{*},\alpha_{2}^{*},\alpha_{3}^{*}$:

(11) $$y_{\Delta }(x)=\alpha_{3}^{*}x^{3}+\alpha_{2}^{*}x^{2}+\alpha_{1}^{*}x+\Delta$$

is vertically shifted by an amount ∆, which is found using the MSAC procedure. The result of this correction procedure is illustrated in Figure 7(b), which shows the fitting of the visible left-boundary (top line) to the mostly occluded right-boundary points (bottom line).

Finally, if any of the consensus sets is empty due to total occlusion of any of the lane's boundaries, an estimation of the lane's position is obtained using the value predicted by an extended Kalman filter (EKF) implemented to track the 3D coordinates of a selection of points belonging to each edge. The details of the EKF implementation can be found in previous work by the authors [52]. The difference in this work with respect to the preliminary research [52] is that the previous filter considers only a simple linear model for the lane boundaries, tracks only the (*ρ*, *θ*) parameters of the lines and does not involve any robust segmentation and parameter estimation procedure as proposed here.

### 3.4. Extended Kalman Filter for Disturbance Mitigation in Lane Estimation and Tracking

In order to reduce the effect of disturbances in the estimation of the lane geometry, we implemented an extended Kalman filter (EKF) [53] due to its good trade-off among the computational efficiency, accuracy and robustness that it provides [54]. Fundamental to the implementation of the EKF are the vehicle's dynamics model characterizing the evolution of its state and the observation's model, which are briefly described next in the context of the proposed approach. For in-depth treatments of vehicle dynamics modeling and state estimation methods, the reader is referred to [53,55,56].

The state of the vehicle is defined in terms of the linear displacements *t_x_*, *t_y_*, *t_z_* along the vehicle-fixed frame and angular displacements *θ_x_*, *θ_y_* and *θ_z_* about the vehicle's reference frame, and their corresponding rates of change, *v_x_*, *v_y_*, *v_z_*, and *ω_x_*, *ω_y_*, *ω_z_*, for the linear and angular velocity, respectively. The inputs to the model can be divided into manipulated and disturbance variables. The manipulated variables are the thrust force *T_f_*, which is adjusted by the throttle controlling the amount of fuel going into the engine, and the steering angle *δ* adjusted by the steering wheel. The steering angle *δ* is the angle between the wheels' rotation plane and the longitudinal axis x*^W^* of the vehicle. The thrust force *T_f_* is assumed to be contained in the plane of rotation of the steered wheels and tangent to the ground plane in the contact pad of the wheels, *i.e.*, we assume a front-wheel-drive model in which *T_f_* acts along the direction *δ* of the steering angle. The disturbance variables in turn can be divided into those representing the vertical geometry of the road described in terms of the slope angle *θ_s_* and the bank angle *θ_b_*, and those representing random disturbances in acceleration due to multiple phenomena, such as wheel slippage and skidding, potholes, speed bumps and humps, raised crosswalks, other vehicle-tire-ground interactions, and aerodynamic drag forces, among others. The slope angle *θ_s_* is the inclination angle of the x^W^-axis with respect to the horizontal inertial plane, *i.e.*, *θ_s_* is the so-called elevation or pitch angle *θ* in Tait-Bryan convention for Euler angles. The bank angle *θ_b_* is the lateral tilt about the x*^W^*, *i.e.*, *θ_b_* is the so-called bank or roll angle in the Tait-Bryan convention for Euler angles. The bank and slope angles change slowly and can be assumed to be piece-wise constant. Moreover, these two variables can be measured with a gyroscope, and therefore, can be input to the model as feedforward (measured) disturbance variables. The linear and angular acceleration disturbances are modeled as zero-mean i.i.d. Gaussian noises *n_x_*, *n_y_*, *n_z_* and *d_x_*, *d_y_*, *d_z_*, respectively. The linear and angular acceleration noises allow to take into account the uncertainty about changes in bank and slope angles of the road, as well as other aspects of the unmodelled dynamics whose effect for practical purposes can be adequately handled by a simplified stochastic model, either because the effect is negligible in comparison with the main model dynamics, or because in practice the cost of accurate parametrization simply outweighs the gains in the accuracy and precision of the state estimation.

Using the above state, input and disturbance variables, the vehicle dynamics is described by the following state-space model formulated in the vehicle-fixed coordinate frame (imagesw) (see [55,56] for detailed standard model derivations):

(12) $$\left[\begin{array}{c} {\dot{t}}_{x}\\ {\dot{t}}_{y}\\ {\dot{t}}_{z}\\ {\dot{\theta }}_{x}\\ {\dot{\theta }}_{y}\\ {\dot{\theta }}_{z}\\ {\dot{v}}_{x}\\ {\dot{v}}_{y}\\ {\dot{v}}_{z}\\ {\dot{\omega }}_{x}\\ {\dot{\omega }}_{y}\\ {\dot{\omega }}_{z} \end{array}\right]=\left[\begin{array}{c} v_{x}\\ v_{y}\\ v_{z}\\ \omega_{x}\\ \omega_{y}\\ \omega_{z}\\ \frac{1}{m}T_{f}\cos(\delta )-\omega_{y}v_{z}+\omega_{z}v_{y}-\frac{c_{x}}{m}v_{x}-g\sin(\theta_{s})+n_{x}\\ -\omega_{z}v_{x}+\omega_{x}v_{z}-g\cos(\theta_{s})\cos(\theta_{b})+n_{y}\\ -\omega_{x}v_{y}+\omega_{y}v_{x}-g\cos(\theta_{s})\cos(\theta_{b})+n_{z}\\ \frac{I_{y}-I_{z}}{I_{x}}\omega_{y}\omega_{z}+\frac{k_{b}}{I_{x}}{\ddot{\theta }}_{b}+d_{x}\\ \frac{I_{z}-I_{x}}{I_{y}}\omega_{x}\omega_{z}+\frac{k_{s}}{I_{y}}{\ddot{\theta }}_{s}+d_{y}\\ \frac{I_{x}-I_{y}}{I_{z}}\omega_{x}\omega_{y}+\frac{1}{I_{z}}T_{f}\sin(\delta )l_{f}+d_{z} \end{array}\right](12)$$

where *m* is the total mass of the vehicle, *g* is the acceleration of gravity, and *I_x_*, *I_y_*, *I_z_* are the inertia moments about the principal axes of the vehicle (the products of inertia *I_xy_*, *I_xz_* and *I_yz_* are neglected because due to the vehicle's symmetry and choice of axes, they are much smaller than the principal moments *I_x_*, *I_y_*, *I_z_*). The parameter *c_x_* is the drag coefficient along the longitudinal axis x*^W^*. Here the model considers that the lateral tire forces of the front and rear wheels, as well as the normal forces of the front and rear wheels, cancel each other when calculating the moment balance about the vehicle axes. This loss of information is taken into account by the angular acceleration disturbance terms *d_x_*, *d_y_* and *d_z_*. Defining a state vector ξ = (*t_x_*, *t_y_*, *t_z_*, *θ_x_*, *θ_y_*, *θ_z_*, *v_x_*, *v_y_*, *v_z_*, *ω_x_*, *ω_y_*, *ω_z_*)*^T^*, inputs vector *v* = (*T_f_*, *δ*, *θ_b_*, *θ_s_*), and disturbance vector *ν* = (*n_x_*, *n_y_*, *n_z_*, *d_x_*, *d_y_*, *d_z_*), it is possible to write the continuous-time state-space model in compact form as ξ˙ = **f** (ξ, *v*, *ν*).

The measurements vector is defined by the vector containing the collection of *N* points **P***_i_* = [*x_i_*, *y_i_*, *z_i_*]*^T^*:

(13) $$\mu (\xi )=\left[\begin{array}{c} {\text{P}}_{1}\\ {\text{P}}_{2}\\ \vdots \\ {\text{P}}_{N} \end{array}\right]$$

segmented from the lane boundaries and expressed in the vehicle-fixed reference frame. The dependence of the points **P***_i_* on the vehicle's state vector ξ results from the fact that the camera frame is rigidly fixed to the vehicle and related to the vehicle frame by a constant coordinate translation and rotation transformation. Therefore, the positions of points **P***_i_* of the lane boundaries (relative to the vehicle frame) change as a result of the vehicle motion according to the well-known equation:

(14) $${\dot{\text{P}}}_{\text{i}}\overset{def}{=}\left[\begin{array}{c} {\dot{x}}_{i}\\ {\dot{y}}_{i}\\ {\dot{z}}_{i} \end{array}\right]=-\left[\begin{array}{c} v_{x}\\ v_{y}\\ v_{z} \end{array}\right]-\left[\begin{array}{c} \omega_{x}\\ \omega_{y}\\ \omega_{z} \end{array}\right]\times \left[\begin{array}{c} x_{i}\\ y_{i}\\ z_{i} \end{array}\right],i=1,2,\ldots ,N$$

The measurement model is simply given by *ζ* = g(ξ, ϵ) = *μ*(ξ) + ϵ, where ϵ ∈ ℝ*^3N^* is a vector of Gaussian random noises accounting for the errors in the segmentation process.

By Euler discretization it is possible to find a recursive approximation of the predicted measurements ζˆ(*k* + 1∣*k*) at instant *k* + 1 in terms of the past measurements and the vehicle's state vector at time *k.* Thus the discretized measurement prediction model can be formulated as:

(15) $$\hat{\varsigma }(k+1|k)=\left[\begin{array}{c} {\text{P}}_{1}(k)-T_{s}V(k)-T_{s}\Omega (k)\times {\text{P}}_{1}(k)\\ {\text{P}}_{2}(k)-T_{s}V(k)-T_{s}\Omega (k)\times {\text{P}}_{2}(k)\\ \vdots \\ {\text{P}}_{N}(k)-T_{s}V(k)-T_{s}\Omega (k)\times {\text{P}}_{N}(k) \end{array}\right]$$

where *T_s_* is the sampling time, *V*(*k*) = [*v_x_*(*k*), *v_y_*(*k*), *v_z_*(*k*)]*^T^* and Ω(*k*) = [*ω_x_*(*k*), *ω_y_*(*k*), *ω_z_*(*k*)] are the vectors of translational and angular velocities of the vehicle at time instant *k*.

The discrete measurement prediction model Equation (15) together with the discretization of the state model Equation (12) can be employed to formulate an extended Kalman filter that allows to reduce the disturbances on the lane geometry estimation produced by the vehicle's motion due to driving maneuvers and vibrations arising mainly from the vehicle-ground interaction. More detailed models of the vehicle's dynamics that consider different aspects of wheel-to-ground contact are possible, see for example [55,56]. Although these models can be used to extend the model employed here, in practice we noticed that the model Equation (12) can be further simplified without severely affecting the accuracy of the results obtained. The main simplifications rely on the fact that the EKF does not lose its capacity to filter high frequency disturbances due to potholes, speed humps, speed bumps, raised crosswalks or small irregularities on the pavement, when the dominating vertical geometry of the road changes at slow rates in comparison with the rate of change in longitudinal and directional variables, *i.e.*, *ω_x_*, *ω_y_* ≪ *ω_z_* and *v_y_*, *v_z_* ≪ *v_x_*. It is to be noted that oversimplified models for the EKF such as the kinematic model proposed by the authors in [52], while helpful in filtering the measurement noise, in practice are less robust to process disturbances, especially when the vehicle moves at higher speeds.

### 3.5. Lane Departure Warning

The lateral distance of the car to the lane boundaries, given by the parameter *α*_0_ of the lane boundary model Equation (10), can be used to compute a measure useful for alerting drivers of an imminent and possibly dangerous unintended line crossing or departure. This measure also takes into account the lateral velocity of the vehicle, which is used to calculate the so-called time-to-lane crossing (TLC) [57]. At a given discrete-time sampling instant *k*, the TLC is computed as:

(16) $${\text{TLC}}_{k}=\frac{d_{k}}{v_{k}}$$

where *d_k_* is the smallest of the lateral distances to the lane's right or left boundaries and *v_k_* is the lateral velocity. For practical purposes, *v_k_* can be adequately approximated by the following moving average of a first-order discrete-time approximation of the lateral distance time-derivative:

(17) $$v_{k}=\frac{1}{N}\cdot \sum_{i=k-N}^{k}\frac{d_{i}-d_{i}-1}{t_{i}-t_{i}-1}$$

where *t_i_* represents the time at sampling instant *i* and *N* is the number of sampling instants considered for averaging.

## 4. Testing Methodology, Experimental Results and Computational Cost

### 4.1. Testing Methodology and Experiment Setup

**Figure 8.**
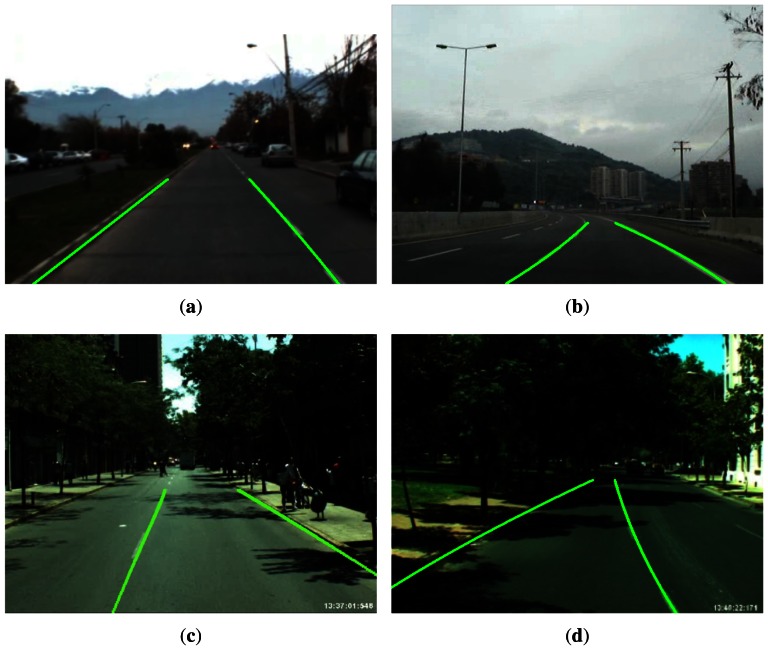
Examples of lane detection and tracking results for the considered scenarios. (**a**) Scenario 1; (**b**) Scenario 2; (**c**) Scenario 3; (**d**) Scenario 4.

The performance of the proposed lane sensing method and departure warning system is evaluated using image sequences acquired during real driving conditions on highways and urban roads in the city of Santiago, Chile. The datasets are grouped in four different scenarios that are summarized in Table 2 (dataset available on-line at: http://ral.ing.puc.cl/datasets/ldw). These scenarios consider different lighting conditions, such as daytime, sunset/dusk and various road conditions with and without lane marks, shadows, and occlusions due to other vehicles, as well as varying road geometry, including straight and curved streets. Curved streets are streets with non-zero curvature in the road plane tangent to the vehicle's wheels. The scenarios considered here include roads with relatively constant slope and bank angles, *i.e.*, their geodesic and torsional curvature (change in slope and bank angles) is negligible in the proximity of the vehicle up to a 100 m. This choice of scenario was motivated by the interest in testing the ability of the proposed approach to measure the road curvature in the plane tangent to the wheels, which is key for lane keeping and departure warning more than the torsional and banking curvatures. Examples showing the projection of the estimated lane boundaries onto the image plane using Equations (6) and (7) are presented in Figure 8 for the four scenarios.

**Figure 9.**
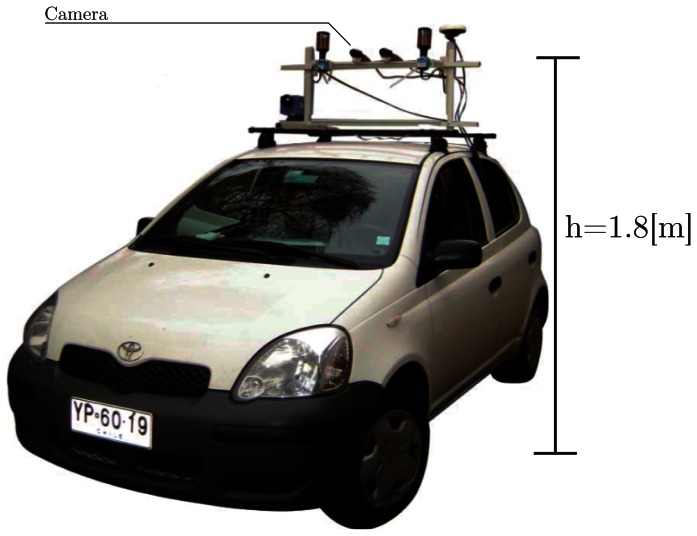
Vehicle for data acquisition.

The video datasets were acquired using a Point Gray^®^ Firewire camera with a 640 × 480 pixels 1/2”CCD and a Tamron varifocal lens with focal distances in the range of 6-12 mm, corresponding to a field view in the range 30.4 ° × 23.1 ° (telephoto) -58.7 ° × 44.4 ° (wide). The camera was rigidly attached to a structure supporting our sensors suite (GPS, thermal, omnidirectional and standard perspective cameras) mounted on the roof of our car test bed, as shown in Figure 9. The camera was located at 1.8 m from the ground with its optical axis pointing to the horizon. With this setup the look ahead distance is about 40 m. The resolution in real world coordinates depends on the image coordinates as can be observed from Equations (4) and (5). The real spatial resolution for an image point with coordinates (*r*, *c*) is given by ∆*_x_* = *x*(*r*, *c*) −*x*(*r* − 1, *c*) and ∆*_y_* = *y*(*r*, *c*) −*y*(*r*, *c* − 1). Considering the closest point to the car (first row *r* = 1, central column *c* = 320), and using Equations (4) and (5), the real spatial resolution with the 640×320 pixels camera and *f* = 9 mm is (∆*_x_*, ∆*_y_*) = (59, 12) mm. This resolution corresponds to that of the pixel *looking* at a point in front of the car at 14 m straight ahead. Similar computations for a point 30 m ahead yield a spatial resolution of (∆_x_, ∆_y_) = (583,54) mm, *i.e.*, the pixel covers approximately 60 cm (depth) by 5 cm (width) of the real world. If a pixel in the horizon is considered, *i.e.*, a pixel with *α_u_* = *α_v_* = 0, then the theoretical spatial resolution of the pixel is infinite, as clearly shown by the denominators of Equations (4) and (5), which become zero, since also *θ*_0_ = 0 for the horizontal camera. As shown by the segmentation results presented in Figures 1, 2 and 6(b), the road segmentation is not perceivably affected by changes in spatial resolution. In the gradient and mean-shift approaches this is explained by the fact that the former only looks at the change in intensities, while the latter performs a sort of clustering by color similarity and spatial proximity. In the case of the approaches using Gabor and GMRF textural features, the relative insensitivity to the spatial resolution of each pixel is achieved by training the classifier with training regions selected from different areas of the road pavement close and far from the vehicle. This ensures that in the operating range of the sensor, the segmentation is performed as correctly as possible, even if in the general case texture is a scale dependent cue. Hence, in general the road segmentation approaches are not significantly affected by the resolution of the imaging sensor. However, it is to be noted that the resolution of the imaging sensor does impose a limit on the accuracy with which the position of the car can be resolved with respect to the edges of the road. If each pixel represents about 5 cm of width in the real world, then in the ideal disturbance-free case with subpixel resolution it would be possible to expect lateral positioning accuracies better than 5 cm. However, interpolation errors and noise make the achievable positioning accuracy worse than the ideal 5 cm. Therefore, regardless of the road segmentation strategy, if higher lateral positioning accuracies are sought, the imaging sensor and the associated optics will have to be replaced accordingly to achieve accuracies that can be estimated using Equations (4) and (5).

### 4.2. Experimental Results: Tracking Accuracy, Misdetections and False Alarms

The accuracy and precision of the lane detection and tracking algorithm is measured in terms of the mean absolute error (MAE), the root mean square error (RMSE) and the standard deviation of the error (σ*_e_*) between the estimated lane geometry and the manually identified curves for the lane boundaries in the analyzed datasets. These results are presented in Table 3. The computation of the MAE, RMSE and standard deviation consider the estimation errors for the left and right lane boundaries simultaneously. The MAE and RMSE errors are computed pointwise, *i.e.*, if the ground truth curve is represented by a set of points (*x_i_*, *y*(*x_i_*)), *i* = 1, 2,…, *N*, sampled from the real lane boundary, and the estimated curve is given by (*x_i_*, *yˆ*(*x_i_*)), *i* = 1, 2,…, *N*, then the estimation error at point *x*_i_ is *e_i_* = *y*(*x_i_*) − *yˆ*(*x_i_*). On the other hand, the reliability of the lane departure warning is measured in terms of false positive (FP) and false negative (FN) rates, as shown in Table 4.

The classic gradient-based approach with steerable filters proposed in [12] is used as a benchmark for evaluating the accuracy and reliability of the proposed approach under the different road segmentation strategies, *i.e.*, using Gabor features, GMRF features and mean-shift clustering. The Gabor-based segmentation employed a bank of eight filters considering wavelengths λ = {8, 4} pixels and orientations *θ* = {0°, 45°, 90°, 135°}; (for a discussion on the selection of adequate parameters for the Gabor filters, see [58]). The GMRF-based segmentation employed the star-like neighborhood *N*(*p*) as recommended in [42]. The mean-shift clustering was performed using a spatial bandwidth *h_s_* = 2, and range parameter *h_r_* = 4, which on average yielded the best segmentation of the road's pavement and shadows under various lighting conditions. The experiments also compare the use of a quadratic lane boundary model in addition to the cubic model Equation (10), and evaluate the benefits of using the EKF, whose parameters can be found in previous work by the authors [52] as mentioned before.

The last three rows of Table 3 summarize the accuracy and precision results for the different lane detection and tracking schemes. The results show a 14.6%-22.6% reduction in RMSE and MAE on average when Gabor filters are employed without the EKF. An additional 7.1%–8.8% reduction on average is possible thanks to the EKF. The GMRF-based segmentation also improves the lane detection, reducing the error by 13.2%-23.9% on average for RMSE and MAE metrics compared with the gradient method. An additional 6.0%-6.9% improvement is achieved using the EFK with the GRMF texture features. The segmentation using the mean-shift algorithm yields the best results with a reduction of 38.0%-38.9% on average in the same metrics with an additional 5.5%–8.1% of reduction in the error when EKF is used. The variance of the error also decreases with any of the three segmentation methods. Once again the mean-shift clustering yields the results with lowest variance.

The difference between the RMSE obtained using the quadratic approximation to the road model and that obtained with the cubic approximation is on average 0.73 ± 0.92 cm. In some scenarios and for some methods, the quadratic model is slightly better than the cubic model, while in other scenarios and with different segmentation approaches, the results show that the cubic model is better by a slim margin. Similarly, the standard deviations of the errors differ by considerably small amounts. Therefore, for practical application the simpler quadratic model may be enough, especially considering that the purpose of the lane departure warning system is to determine whether the vehicle is at risk of leaving the lane given its current speed and the road's curvature. Using a quadratic road model, *i.e.*, *α_3_* = 0 in Equation (10), the maximum (worst case) curvature is precisely given by the second order derivative of the quadratic polynomial, *i.e.*, the maximum curvature is 2*α_2_*. This maximum curvature provides a measure of the amount of steering that the driver should be applying to correctly handle the curve.

The last column of Table 4 summarizes the reliability of the lane departure warning system in terms of FP and FN rates. The different approaches are evaluated using a threshold of *δ* = 1.0 *s* for the minimum allowable time to lane crossing (TLC) before triggering an alarm of unintended lane departure. In comparison with the gradient-based benchmark method, a decrease in the FP and FN rates of 49.5% and 52.3% is achieved on average using Gabor filters, with an additional decrease of 4.7% and 4.6% on both metrics when also the EKF is used. The GMRF-based segmentation yields a decrease in the rate of FP and FN of 34.8% and 57.1% on average, and an additional 11.1% and 7.2% when also the EKF is employed. Finally, when comparing the mean-shift clustering approach to the gradient-based benchmark method, a larger decrease in the FP and FN rates of 55.3% and 65.5% is possible, with additional relative reduction of 2.9% and 2.1% with the EKF. The lane departure warning estimation has no significant impact on the processing times, since it relies on the results of the lane detection and tracking step to compute simple mathematical operations involved in the TLC measure.

### 4.3. Computational Cost

The theoretical computational complexity for each of the road segmentation approaches is summarized in Table 5 (fourth column). The computational complexity is a function of (i) the size of the input and parameters that predefine certain volume of computations; and (ii) the type of computational process or mathematical operation involved. The input data and parameter characteristics of each road segmentation strategy are shown in the second column of Table 5, while the main computational process behind each approach is described in the third column. In the case of the gradient-based and Gabor-based approaches, the main operation that contributes to the algorithms' complexity is the convolution of the image with each filter response. It is possible to show (see for example [59]) that the standard implementation of the convolution of a filter mask of size *P* x *Q* with an image of size *M* x *N* is *O*(*PQMN*), and therefore, the main difference in computational complexity between the gradient and the Gabor approach is the number of times *D* and *B* that the convolution must be repeated, respectively. On the other hand, the computational complexity of the GMRF approach arises from the solution of a linear least squares problem involving matrix inversion and multiplication steps. Thus, while the convolution of two sets with *n* data points has a complexity *O*(*n^2^*), the matrix inversion of an n-dimensional matrix has a complexity *O*(*n^3^*) using standard Gauss-Jordan elimination. Due to the connection between matrix multiplication and matrix inversion, the number of operations for the solution to the least squares problem could be reduced with Strassen or Coppersmith-Winograd matrix multiplication algorithms [60], but with substantial bookkeeping overhead that makes these algorithms not practical unless *n* ≫ 100 [61], and thus they are not implemented here. Since the GMRF approach takes the expected value of the solution to a linear least squares problem in a neighborhood of size *n*, the actual cost of GMRF becomes *O*(*n*^4^). If the size of the GMRF neighborhood is *P* × *P* and is assumed symmetric, then *n* = *P*^2^/2, so the cost at each location becomes at least of the order *O*(*P*^8^/16). Finally, the complexity of the mean-shift approach stems from the neighborhood query for each of the *n* points in the dataset, and therefore, if the points belong to a *d*-dimensional dataset and *N* iterations are performed, the complexity of the naive method is *O*(*n*^2^*dN*) [62].

The comparison of the computational complexities at the practical level is not simple because there are many implementation aspects that are specific to each approach, for example, the computation neighborhood (subwindow block) centered at each pixel must be of a different size in each approach in order to achieve the best segmentation. On the other hand, while Gabor and GMRF require the training of a classifier (which is done off-line only once), the approaches based on the gradient and mean-shift computation do not require the construction of a classifier. Hence, there are other factors, such as the choice of classifier, that can affect the computational cost of the approach in ways that are far from subtle. However, a gross estimate of the relative computational complexity can be obtained with the simplifying assumption that the computation neighborhood of size *P* x *Q* centered at each pixel of interest is (i) square and (ii) proportional to the image size by a factor *β*, *i.e.*, *P* = *Q* = *βM* = *βN*. With this assumption, the computational complexity for each of the four methods in Table 5 will be given respectively by *O*(*β*^2^*M*^4^*D*), *O*(*β*^2^*M*^4^*B*), *O*(*β*^9^*M*^10^), *O*(*αβ*^4^*M*^4^(*C* + 2)*K*) < *O*(*αβ*^5^ *M*^5^), for the gradient, Gabor, GMRF, and mean-shift approaches. If additionally it is assumed that *β* = 10^−1^, *M* = 10^3^, then the order of floating point operations will be respectively, 10^10^*D*, 10^10^*B*, 10^21^, and 10^9^. For *D* = 3 (three directions) and *B* = 16 (a Gabor filter bank of dimension 16), the Gabor-based approach should take about 5 times more to compute than the gradient-based approach, the GMRF-approach would be in theory at least 10^10^ times slower than the gradient-based approach, while the mean-shift approach would be similar or faster to compute than the gradient-based method. In practice, for our implementation on a computer with a 2 GHz Intel^®^ Core^TM^ 2 Duo CPU, it was observed that the road segmentation with the Gabor filter takes 4 times longer to compute than the basic gradient-based method, as shown by the results summarized in Table 6. This result is in close agreement with the theoretical estimates. However, contrary to the theoretical predictions, the mean-shift method was 2 times slower than the gradient-based approach, but as it yields processing rates of 5.6 fps on average on a standard CPU, it should be suitable for real-time computation on dedicated hardware. On the other hand, the GMRF-based approach implemented with an eleven-dimensional feature vector was 300 times slower than the basic gradient-based approach. This is significantly better than the cost that can be predicted from the theoretical complexity estimates, but due to the large processing time of 26.7 seconds per frame that it required in the experiments, the GMRF-based approach seems unfit for practical application to driver assistance using the currently available embedded-computing technology for vehicles.

The computational complexities presented in Table 5 do not include the cost of the MSAC variant of the RANSAC method for parameter estimation nor the cost associated to the extended Kalman filter, because the additional cost of these stages is the same regardless of the choice of pavement segmentation strategy. Moreover, on average these steps add less than 10 ms to the computation of each frame. However, the cost of the MSAC and extended Kalman filter is included in the experimental measurements of the computational cost presented in Table 6. For a discussion on the theoretical complexity of the RANSAC approach and the extended Kalman filter, the reader is referred to [63–66].

## 5. Conclusions

This work proposed and assessed the effectiveness of using texture based segmentation or color-clustering methods for robust lane detection and tracking. The results showed that the road can be extracted robustly using the mean-shift method for clustering regions of similar color despite shadows and other variability in road materials, structure and illumination (cf. results highlighted in Table 3). Texture features also improved the effectiveness of the classic methods based on edge-detection. However, obtaining textures is computationally demanding when compared with the mean-shift color clustering, and therefore textures are less adequate for applications that require high frame processing rates.

This work also proposed an approach to robustly determine the road geometry despite the presence of occlusions and shadows, which typically introduce discontinuities in the extracted lane boundaries. To achieve this improvement the standard clothoid road model is approximated by its power series expansion of order three. Obtaining the parameters of the cubic polynomials corresponding to the correct lane geometry can be carried out successfully despite outliers generated by such disturbances as shadows and occlusions using the MSAC variant of the RANSAC robust parameter estimation approach. The experiments showed that a quadratic model is enough for practical purposes, and that the cubic polynomial model does not yield significantly better results.

The results confirmed that further improvements to lane sensing and departure warning are possible using an Extended Kalman filter to predict the lane location on subsequent frames whenever there are severe occlusions. It is to be noted that the proposed approach performs the lane tracking in the 3D space of motion using a single camera and not in the 2D image space as most of the existing lane tracking approaches do. This has the advantage of enabling a more robust estimation of the road geometry and the car's position relative to the lane. Tracking points in 3D space requires the inverse perspective mapping of points in the 2D image space back to the 3D space, which is shown to be possible thanks to the knowledge of the height and tilt angle at which the camera is mounted on the roof of the vehicle; however, in general the inverse perspective projection would require a multi-view approach using more than one camera. Ongoing research is concerned with the application of the proposed lane sensing approach, and in particular the analysis of lane outliers, to the challenging problem of intersection recognition. Also the ongoing research considers the integration of LADAR measurements to obtain an accurate 3D model of the road geodesic and torsional curvature using a standard Darboux frame, thus to overcome some limitations imposed by the locally flat road model in the back projection of image points to the 3D space for points far in the horizon.

## Figures and Tables

**Table 1.** Comparison of a selection of lane detection and tracking approaches published in 2004–2012 (see the recent surveys [10,12] for in-depth discussions about foundational and influential developments in lane detection).

**System**	**Use**^a^	**Road Model**	**Feature Extraction**	**Postprocessing**	**Tracking**^b^	**Evaluation** *E_i_***/Frames/ fps/Positioning Accuracy**^c^
Wijesoma *et al.* (2004) [17]	C	Quadratic curve on planar road	2D Ladar measurements	Thresholding of measured data	EKF	*E*_2_/N.A./10/N.A.
Springrobot (2004) [33]	A B	Circular arcs on planar road	Edge elements	Adaptive Randomized Hough transform	N.A.	*E*_1_/N.A./N.A./N.A.
Wang *et al.* (2005) [34]	B	Polynomial curve planar road	Edge elements	GPS measures integration	KF	*E*_1_/N.A./N.A./N.A.
Kim (2008) [35]	C	Cubic-spline curve on planar road	Lane-marking classifiers	RANSAC curve fitting	PF	*E*_1_/1800/10/N.A.
Danescu *et al.* (2009) [36]	B	3D model considering vertical and horizontal curvature	Image gradient from stereo cameras	Probability density estimation	PF	*E*_1_/4200/10/N.A.
Amditis *et al.* [37]	B	Clothoid on planar road	Edge elements	Hough Transform and GPS data fusion	EKF	*E*_1_/N.A./N.A./N.A.
Cheng *et al.* (2010,2006) [28,38]	C	Piecewise linear	Color analysis of lane marks in structured roads and mean-shift clustering in unstructured roads	External vehicle elimination procedures	Lane-coherence-verification	*E*_1_/6000/10/97.4%
Ruyi *et al.* (2011) [31]	B	Locally linear on planar road	Edge elements	Edge blobbing and distance transform operations	PF	*E*_1_/1224/10/N.A.
Guo *et al.* (2012) [11]	B	Planar road with segmented 3D structure and no explicit curvature model	Stereoscopy using Markov random fields	Textureless regions elimination	N.A.	*E*_1_/15000/15/N.A.

a A: Autonomous vehicle control, B: Driver assistance, C: Unspecified;

b *E*_1_: road images, *E*_2_: controlled scenario, frames: capture length, fps: data capture rate;

c KF: Kalman Filter, EKF: Extended Kalman Filer, PF: Particle Filter, N.A.: Not Available.

**Table 2.** Scenarios for the performance evaluation of the lane sensing and departure warning system.

**Scenario**	**Road Geometry**	**Lane Marks Quality**	**Lighting Condition**	**Frames**
Lane boundaries detection and tracking system				

1	Straight	Fair	Daytime, no shadows	340
2	Straight and curved	Fair	Daytime, no shadows	350
3	Straight	Poor	Sunset, shadows	410
4	Straight and curved	Poor	Dusk, shadows	380
Lane departure warning system				

1	Straight	Fair	Daytime, no shadows	340
2	Straight and curved	Fair	Sunset, shadows	280
3	Straight and curved	Poor	Dusk, shadows	300

**Table 3.** Lane detection and tracking results.

		Method													

		G		Gabor Filter Segmentation				GMRF Segmentation				Mean-Shift Clustering			

				G		G + EKF		G		G + EKF		G		G + EKF	

		Q	C	Q	C	Q	C	Q	C	Q	C	Q	C	Q	C
S_1_	M_1_	12.12	13.21	10.11	9.81	9.21	9.31	11.13	12.04	11.01	10.98	9.11	9.51	8.51	8.62
	M_2_	13.33	14.81	11.15	10.15	9.51	10.11	13.01	13.05	12.05	12.33	9.47	10.03	9.02	9.11
	M_3_	3.21	3.92	2.91	3.01	2.51	3.13	4.01	3.98	3.68	3.56	2.19	2.52	2.21	2.41

S_2_	M_1_	13.62	14.11	9.81	9.71	9.11	9.12	11.51	10.65	11.21	10.45	9.31	9.61	8.05	8.42
	M_2_	14.77	14.96	10.52	10.33	9.51	10.11	12.01	11.31	10.56	10.87	9.79	10.03	9.12	9.21
	M_3_	3.41	3.99	2.16	2.81	2.01	2.38	5.01	4.67	4.51	4.01	2.11	2.42	2.11	2.19

S_3_	M_1_	15.62	19.11	13.81	13.93	12.19	12.82	14.21	13.56	14.11	13.52	9.41	10.11	8.91	9.43
	M_2_	16.75	20.96	14.51	15.11	13.16	13.21	15.32	13.89	14.54	15.02	10.09	11.31	9.52	9.81
	M_3_	6.11	7.97	4.16	3.81	3.14	3.28	4.71	6.11	4.65	3.98	2.51	2.28	2.19	2.25

S_4_	M_1_	18.21	18.41	17.13	18.95	16.09	14.81	14.65	14.67	13.67	14.01	9.11	10.41	8.21	8.72
	M_2_	19.58	19.96	18.21	19.15	17.66	15.11	15.55	15.51	14.32	14.33	10.01	11.81	9.33	9.29
	M_3_	7.91	8.12	5.61	5.18	5.04	4.33	4.56	5.21	4.19	4.97	2.11	2.18	2.10	2.17

	$\bar{M_{1}}$	14.89	16.21	12.71	13.10	11.65	11.51	12.87	12.73	12.50	12.24	9.23	9.91	8.42	8.79
	$\bar{M_{2}}$	16.10	17.67	13.61	13.68	12.46	12.13	13.97	13.44	12.86	13.13	9.84	10.79	9.25	9.36
	$\bar{M_{3}}$	5.16	6.00	3.71	3.70	3.17	3.28	4.57	4.99	4.25	4.13	2.23	2.35	2.15	2.25

*S_i_* : Scenario *i* = {1, 2, 3, 4}; *M*_1_: MAE [cm]; *M*_2_: RMSE [cm]; *M*_3_: σ*_E_* [cm]; G: Gradient features extraction; EKF: Extended Kalman Filter; Q: RANSAC quadratic curve fitting; C: RANSAC cubic curve fitting.

**Table 4.** Lane departure warning results.

**Method**	**Metric[% ]**	**Scenario 1**	**Scenario 2**	**Scenario 3**	**Mean**
Gradient	FP	8.12	11.81	12.41	10.78
	FN	12.43	13.44	9.92	11.93

Gabor Filter Segmentation	FP	5.76	4.44	6.11	5.44
	FN	5.11	6.31	5.65	5.69

Gabor Filter Segmentation and EKF	FP	5.16	4.09	5.53	4.93
	FN	4.45	5.71	5.27	5.14

GMRF Segmentation	FP	6.44	7.94	6.67	7.02
	FN	7.12	4.12	4.11	5.12

GMRF Segmentation and EKF	FP	5.23	6.23	6.01	5.82
	FN	6.51	3.25	3.01	4.26

Mean-shift Clustering	FP	4.01	5.32	5.13	4.82
	FN	4.21	3.31	4.84	4.12

Mean-shift Clustering and EKF	FP	3.71	4.88	4.91	4.50
	FN	4.01	3.21	4.39	3.87

**Table 5.** Computational complexity of the different approaches.

**Approach**	**Input**	**Complexity Sources**	**Computational Complexity**
Gradient	Grayscale image of *M* x *N* pixels *P* x *Q* convolution mask Gradient Orientations *D*	Convolution of a bank of *D* oriented Laplacian of Gaussian filter responses of size *P* x *Q* with the image.	*O*(*PQMND*)
Gabor segmentation	Grayscale image of *M* x *N* pixels *P* x *Q* convolution mask λ wavelengths, *θ* orientations, and *ϕ* phases	Convolution of a bank of *B* Gabor filter responses of size *P* x *Q* with the image, with *B* = λ • *θ*· *ϕ*.	*O*(*PQMNB*)
GMRF segmentation	Grayscale image of *M* x *N* pixels *P* x *P* symmetric neighborhood with *R* = *P*^2^/2 + 1 parameters	Linear least squares estimation involving a *R* x *R* matrix inversion with complexity *O*(*R*^3^) and a matrix-vector multiplication (*R* x *R*) · *R* with complexity *R*^3^ repeated *R* times on each pixel neighborhood and, then repeated *M N* times for each image pixel, *i.e.*, *O*(*R* · (2*R*^3^)) for each pixel.	*O*((*P*^8^/16)*M N*)
Mean-shift clustering	Color image of *M* x *N* pixels and *C* channels Maximum mean shift iterations *K*	In the naive implementation, query all the points in the dataset (neighborhood) around the current point to check if the kernel of each point in the dataset covers the current point. If a tessellation strategy is implemented, an improvement factor *α*, with 0.05 < *α* < 0.1, is possible.	*O*(*α* · (*M N*)^2^ (*C* + 2)*K*)

**Table 6.** Comparison of the computational time of lane detection and tracking.

**Approach**	**Average Processing Time Per Frame (s)**	**Frames Per Second**
Gradient	0.09	11.1
Mean-shift	0.18	5.6
Gabor filters	0.34	2.9
GMRF	26.7	0.04
